# Overexpression of *BnaMATE43b* Improves Resistance to Aluminum Toxicity and Identification of Its Upstream Transcription Factors in Rapeseed (*Brassica napus* L.)

**DOI:** 10.3390/plants15020338

**Published:** 2026-01-22

**Authors:** Xiaojun Xiao, Huiwen Zhou, Paolan Yu, Wei Zheng, Depeng Han, Lei Yang, Zhexuan Jiang, Yewei Cheng, Yazhen Li, Tianbao Huang, Wen Xiong, Xiaoping Huang, Ming Chen, Xiaosan Liu, Meiwei Zhang, Yingjin Huang, Qinghong Zhou

**Affiliations:** 1 Key Laboratory of Crop Physiology, Ecology and Genetic Breeding, Ministry of Education, Agronomy College, Jiangxi Agricultural University, Nanchang 330045, China; xiao850908@163.com; 2 Jiangxi Institute of Red Soil and Germplasm Resources, Nanchang 330046, China; 15270904956@163.com (P.Y.); zw07917043299@163.com (W.Z.); handepeng1113@163.com (D.H.); jxmhsyl@126.com (L.Y.); jzxhrs2510@163.com (Z.J.); 18407851644@163.com (Y.C.); liyazhen626@163.com (Y.L.); htb1234@163.com (T.H.); 15179121371@163.com (W.X.); mingchen8454@126.com (M.C.); lxs_0704@163.com (X.L.); m18235413715@163.com (M.Z.); 3 Institute of Jiangxi Oil-Tea Camellia, College of Pharmacy and Life Science, Jiujiang University, Jiujiang 332005, China; hwzhou0320@jju.edu.cn; 4 College of Life and Environmental Sciences, Hangzhou Normal University, Hangzhou 311121, China; xphuang@hznu.edu.cn

**Keywords:** *Brassica napus* L., overexpression of *BnaMATE43b*, aluminum tolerance, metabolic pathway

## Abstract

The multidrug and toxic compound extrusion (MATE) protein plays a crucial role in mediating plant responses to aluminum (Al) toxicity. The key candidate gene *BnaMATE43b* related to Al toxicity stress in rapeseed was identified using GWAS and transcriptome analysis. In this study, the *BnaMATE43b* gene was cloned and functionally characterized in rapeseed. Compared with wild-type rapeseed (WT), the *BnaMATE43b* overexpression lines (OE) demonstrated stronger aluminum tolerance, specifically manifested in higher relative elongation of taproots (RETs) and relative total root length (RTRL); under Al toxicity stress, the enzyme activities (SOD and POD) and root activity were significantly increased in the OE lines, whereas the MDA content and relative electrical conductivity were reduced in rapeseed root. Further transcriptome analysis of OE-3 showed that the differentially expressed genes (DEGs) were mainly enriched in zeatin biosynthesis (map00908), glucosinolate biosynthesis (map00966), phenylpropanoid biosynthesis (map00940), and ascorbate and aldarate metabolism (map00053). In addition, the yeast cDNA library of rapeseed was constructed, and twenty-two candidate upstream transcription factors (UTFs) of *BnaMATE43b* were screened; furthermore, four candidate UTFs were obtained through one-on-one interaction validation and luciferase assays, comprising three bHLH transcription factors (*BnaA02g28220D*, *BnaA06g07840D*, and *BnaA08g24520D*) and one ERF transcription factor (*BnaA05g23130D*). Collectively, these results suggest that *BnaMATE43b* could improve Al tolerance in rapeseed by mediating antioxidant enzyme activities and the related metabolic pathway, while the obtained UTFs lay the foundation for further analysis of the gene regulatory network under Al toxicity stress.

## 1. Introduction

Rapeseed (*Brassica napus* L.) is an important source of vegetable oil, and the Yangtze River basin is the main rapeseed production area in China. Unfortunately, most of the rapeseed in the Yangtze River basin grows in acidic red soil areas with a pH lower than 5.5 [1,2]. In such soil, exchangeable aluminum (Al) is released from relatively stable silicates or oxides, causing Al toxicity stress [3,4]. Several reports have revealed that Al toxicity induces MDA and ROS generation, and the growth of rapeseed roots is significantly inhibited under Al toxicity stress, ultimately leading to a significant decrease in yield [1,5,6].

The responses of plants to Al toxicity involve complex biological processes, involving numerous genes and metabolic pathways [6,7,8]. For instance, the aluminum-activated malate transporter (ALMT) and multidrug and toxic compound extrusion (MATE) families of transporters mediate the transport of citrate acid, which chelates Al ions to alleviate Al toxicity-related damage [9,10]. Research has shown that overexpression of *ZmMATE6* can improve the release of citrate from roots compared with controls, which may reduce the accumulation of Al ions in the root tissues, resulting in significant resistance to Al toxicity stress [11]. Heterogeneous expression of *BoMATE* in *Arabidopsis thaliana* and *GmMATE13* in soybean hairy roots enhanced Al tolerance by increasing citrate efflux [12,13]. In addition, MATE regulated root development by modulating auxin levels, which simultaneously regulated Al tolerance in mutants [14]. BiMATE can improve tolerance to acid/aluminum stress during the seedling stage, which may be related to the increase in antioxidant enzyme activity [15]. These results establish MATE proteins as essential components in plant Al stress response, making it imperative to further explore their functional diversity and genetic regulation. Transcription factors, such as *ZFP36*, *SbXTH7*, and *ANAC070*, play an important role in mediating root development in plants under Al toxicity stress [16,17,18]. Moreover, the expression of chalcone synthase and isoflavone synthase was shown to be highly enhanced by overexpression of *GmSTOP1-3*, which increased the contents of naringenin chalcone, naringenic, and genistein in soybean roots under Al toxicity stress; ultimately, the Al tolerance of soybean improved [19]. In a plant’s response to Al toxicity stress, STOP1 was shown to be a core transcription factor that can regulate the expression of numerous genes (e.g., *MATE*, *ALMT*, *RAE1*), thereby regulating the Al tolerance of plants [7,19,20]. *GmABR1* may jointly regulate plant resistance to Al stress through genes (*AtMATE*) related to Al stress response and ABA response pathways [21]. The GmWRKY21 transcription factor may promote tolerance to aluminum stress mediated by pathways regulating the expression of the acidic aluminum stress-responsive genes and abiotic stress-responsive genes (e.g., *MATE*, *ALMT*, *ALS3*) [22]. It remains to be further investigated whether there are other transcription factors regulating the expression of *MATE*. The yeast one-hybrid (Y1H) assay is a direct technique for screening upstream transcription factors [23,24], and this technique is widely used in hot pepper [24], wheat [25], and *Arabidopsis* [26].

In our previous studies, *BnaA03g30320D* was identified in relation to Al tolerance in rapeseed based on the integration of genome-wide association analysis (GWAS) and transcriptome analysis (RNA-seq) [27]. This gene encodes protein detoxification 43 belonging to the MATE family, named *BnaMATE43b* [28]. Then, the function of *BnaMATE43b* under Al toxicity stress was verified through overexpression in rapeseed, and the phenotype, physiological response, and transcriptome associated with the responses of overexpression lines (OEs) to Al toxicity were analyzed. This study provides a functional analysis to expand our understanding of the physiological response of *BnaMATE43b* under Al toxicity stress.

## 2. Results

### 2.1. Expression Level Detection, Cloning, and Genetic Transformation of BnaMATE43b

**Figure 1:**
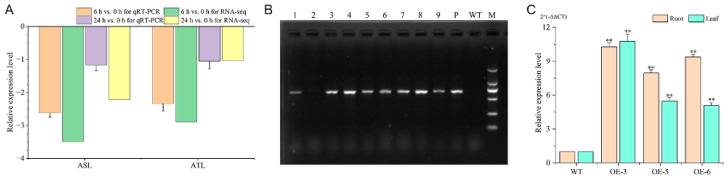
Expression of *BnaMATE43b* in rape root and transgenic-positive plants. (**A**) Expression of *BnaMATE43b* in rape root based on qRT-PCR and RNA-seq. (**B**) Positive identification of transgenic plants. Lane 1 to Lane 9 represent 9 genetically transformed plants; Lane 10 is the recombinant plasmid pCAMBIA1301-BnaMATE43b; Lane 11 is the acceptor material Westar; Lane M is the ≤2 kb marker (2000, 1000, 750, 500, 250, 100 bp). (**C**) Relative expression level of the overexpressing *BnaMATE43b* plant. Significance analysis was performed using a *t*-test (** *p* ≤ 0.01).

The qRT-PCR results showed that the expression patterns of *BnaMATE43b* in both the Al-tolerant inbred line (ATL) and the Al-sensitive inbred line (ASL) were basically consistent with RNA-seq (Figure 1A). The recombinant plasmid pCAMBIA1301-BnaMATE43b was transformed into wild-type rapeseed Westar (WT). Eight T0-positive plants were confirmed (Figure 1B). Ultimately, three T3 transgenic lines (OE-3, OE-5, and OE-6) displayed higher expression levels in root and leaf compared with those of WT (Figure 1C).

### 2.2. Phenotype Characterization of Overexpressing BnaMATE43b

**Figure 2:**
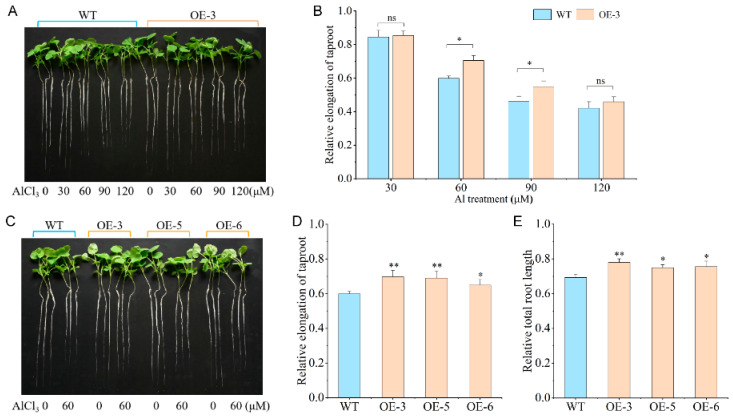
The phenotype traits in WT and OE lines under Al toxicity stress. (**A**) The WT plants and OE-3 under 0, 30, 60, 90, and 120 μmol·L^−1^ AlCl_3_ treatments. (**B**) The RET statistics of WT and OE-3 under 0, 30, 60, 90, and 120 μmol·L^−1^ AlCl_3_ treatments. (**C**) WT and OE lines under 60 μmol·L^−1^ AlCl_3_ treatment. (**D**) The RET statistics of WT and OE lines under 60 μmol·L^−1^ AlCl_3_ treatment. (**E**) The RTRL statistics of WT and OE lines under 60 μmol·L^−1^ AlCl_3_ treatment. Significance analysis was performed using a *t*-test (ns *p* > 0.05, * *p* ≤ 0.05, ** *p* ≤ 0.01).

As OE-3 showed a higher expression level, it was used as the material to identify the appropriate concentration of AlCl_3_ for plant phenotype and physiological response. Compared with the control, there was no significant difference for the relative elongation of taproots (RETs) of WT and OE-3 under 30 μmol·L^−1^ and 120 μmol·L^−1^, but there was a significant difference under the 60 μmol·L^−1^ and 90 μmol·L^−1^ AlCl_3_ treatments. The taproot elongation values of OE-3 were 8.30 cm and 6.39 cm under the 60 μmol·L^−1^ and 90 μmol·L^−1^ treatments, respectively. The RET was improved by 17.65% for the 60 μmol·L^−1^ treatment and 18.97% for the 90 μmol·L^−1^ treatment (Figure 2A,B). Combined with taproot elongation and the RET, the 60 μmol·L^−1^ AlCl_3_ treatment was used to further research phenotypic identification and physiological response.

There was no significant difference in the taproot length and total root length between the WT and OE lines (OE-3, OE-5, and OE-6) under the control treatment (0 μmol·L^−1^ AlCl_3_). After 7 days of the 60 μmol·L^−1^ AlCl_3_ treatment, compared with WT, the RET and relative total root length (RTRL) in the OE lines were significantly increased by an average of 13.41% and 10.1%, which suggests that overexpression of *BnaMATE43b* could improve the Al tolerance of rapeseed (Figure 2C–E).

### 2.3. Physiological Response of Overexpressing Lines to Al Treatment

**Figure 3:**
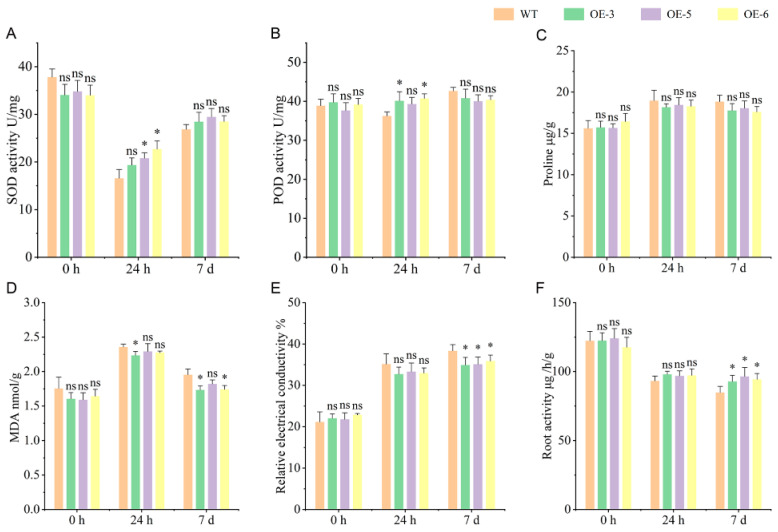
The physiological response in the OE lines and WT under Al toxicity stress. (**A**) The SOD activity in rapeseed root. (**B**) The POD activity in rapeseed root. (**C**) The content of proline in rapeseed root. (**D**) The content of MDA in rapeseed root. (**E**) The relative electrical conductivity in rapeseed root. (**F**) The root activity in rapeseed root. Significance analysis was performed using a *t*-test (ns *p* > 0.05, * *p* ≤ 0.05).

Under the control treatment (0 h), no significant differences were observed in all measured physiological indices between the OE and WT lines. Interestingly, distinct differences emerged between the OE lines and WT after exposure to 60 μmol·L^−1^ AlCl_3_ stress for both 24 h and 7 days (Figure 3).

The superoxide dismutase (SOD) activity in WT was significantly lower than that in the OE lines after 24 h of Al treatment, but this difference disappeared after 7 days of treatment (Figure 3A). A similar trend was observed for peroxidase (POD) activity, which increased moderately and remained stable across treatments (Figure 3B). The content of proline increased when the seedlings were exposed to a nutrient solution with 60 μmol·L^−1^ AlCl_3_ (Figure 3C). The MDA content increased under Al treatment, with a significant difference between the WT and OE lines after 7 days of treatment (Figure 3D). Relative electrical conductivity showed an inverse trend with root activity, increasing under Al toxicity stress (Figure 3E). The OE lines exhibited significantly higher root activity than WT, with a marked difference in MDA content between the WT and OE lines after 7 days of treatment (Figure 3F).

### 2.4. Transcriptome Analysis of Overexpressing BnaMATE43b

**Figure 4:**
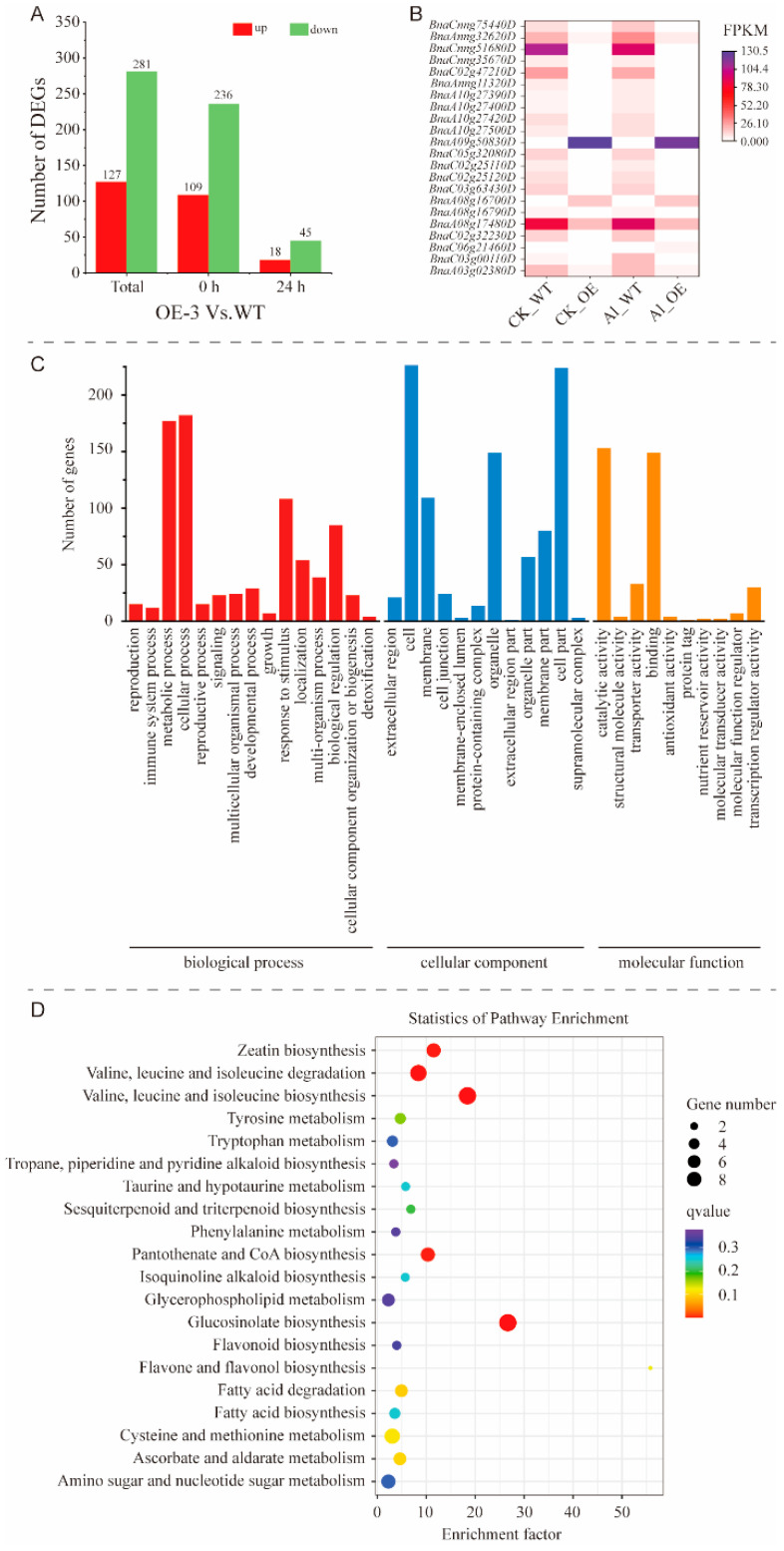
Analysis of the DEGs at 24 h and 0 h under Al toxicity treatment of OE-3 and WT. (**A**) The distribution map of DEGs. (**B**) Heat map of some common DEGs between WT and OE-3 under Al toxicity stress. (**C**) GO enrichment of the DEGs. (**D**) KEGG enrichment of the DEGs.

Compared to the WT (0.532 ± 0.01), the RET of OE-3 (0.613 ± 0.034) was significantly increased by 15.23% under Al treatment for 24 h. For RNA-seq, 255 and 249 million clean reads in the WT and OE-3 were obtained and mapped to a reference genome after data filtering, respectively (Supplementary Table S1). A total of 408 DEGs were found between OE-3 and WT, comprising 127 upregulated DEGs and 281 downregulated DEGs. For the control treatment (0 h), 109 DEGs showed upregulation and 236 DEGs showed downregulation in OE-3 compared with WT. In total, 18 DEGs showed upregulation and 45 DEGs showed downregulation at 24 h (Figure 4A, Supplementary Tables S2 and S3). Among these DEGs, 22 common DEGs were screened between OE-3 and WT both at 0 h and 24 h (Figure 4B).

Gene Ontology (GO) analysis revealed that 386 of the 408 DEGs were significantly enriched in various terms. These DEGs were mainly enriched in biological processes, including metabolic process (177) and cellular process (182), cellular components, such as cell (226) and cell part (224), and molecular functions, including catalytic activity (153) (Figure 4C). Each GO term contains one or multiple genes with known functions, and some genes are enriched in multiple GO terms (Supplementary Table S4). Kyoto Encyclopedia of Genes and Genomes (KEGG) analysis indicated that 86 DEGs were enriched in 64 pathways, including zeatin biosynthesis (map00908); valine, leucine, and isoleucine degradation (map00280); valine, leucine, and isoleucine biosynthesis (map00290); pantothenate and CoA biosynthesis (map00770); glucosinolate biosynthesis (map00966); plant hormone signal transduction (map04075); phenylpropanoid biosynthesis (map00940); and ascorbate and aldarate metabolism (map00053) (Figure 4D, Supplementary Table S5).

### 2.5. Construction of Yeast cDNA Library of Rapeseed

**Figure 5:**
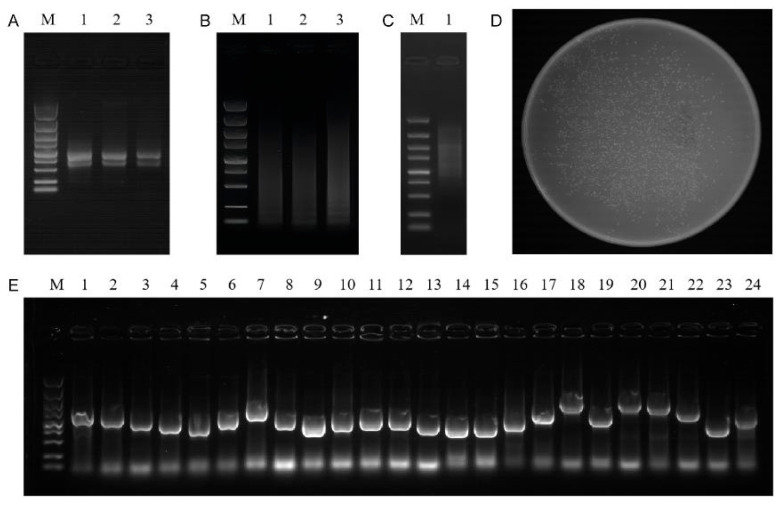
The construction of a normalized cDNA library. (**A**) Total RNA detection. Lane M is the 5 kb marker (5000, 3000, 2000, 1500, 1000, 750, 500, 250, 100 bp) (the same applies to the following). Lanes 1, 2, and 3 are the total RNA repeatedly extracted with different biological repetitions. (**B**) The synthesis of the double-stranded cDNA. Lane M is the 5 kb marker. Lane 1 is P1-F/P4-R-amplified ds cDNA; Lane 2 is P2-F/P4-R-amplified ds cDNA; Lane 3 is P3-F/P4-R-amplified ds cDNA. (**C**) Lane M is the 5 kb marker. Lane 1 is the purification results of three ds cDNA mixes. (**D**) Lane M is the 5 kb marker. Growth of bacteria in the yeast library in 1/10,000 dilution. (**E**) In total, 24 clones were randomly selected to identify the recombination rate and the average length of the inserted fragments using colony PCR.

Agarose gel electrophoresis confirmed the integrity of the total RNA, with clear bands corresponding to the 28S and 18S rRNA subunits (Figure 5A). The RNA selected from Lane 2 was used to construct the normalized cDNA library and synthesize cDNA, which showed diffuse bands (Figure 5B). After normalization and elimination of small fragments, the double-stranded cDNA showed diffuse bands and no obvious bright bands (Figure 5C). Finally, about 1420 clones containing secondary library plasmids were grown in Petri dishes with a titer of 1.6 × 10^8^ CFU/mL (Figure 5D). The length of the inserted fragments was mainly 750 bp~2000 bp, and the combination rate reached 100% (Figure 5E).

### 2.6. Screening the Potential Upstream Transcription Regulators of BanMATE43b

**Figure 6:**
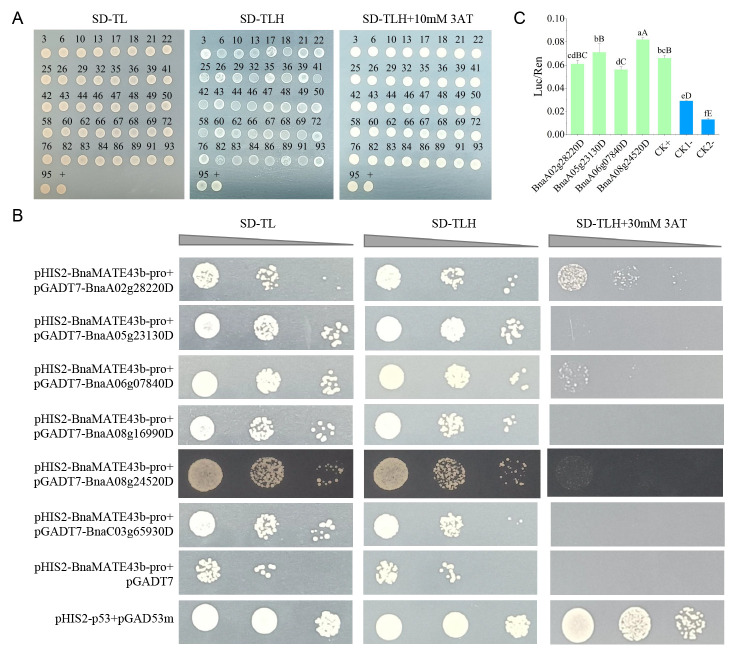
Screening of factors regulating *BnaMATE43*. (**A**) The rotary verification + is pHIS2-p53 + pGAD53m in the positive control group. (**B**) The result of point-to-point interaction verification. (**C**) The dual luciferase reporter assay of the *BnaMATE43* promoter (small letters *p* ≤ 0.05, big letters *p* ≤ 0.01).

Based on Y1H, 96 monoclones were randomly selected from the transformation plate. Among these, 41 single clones were obtained using BLAST 2.14.0 analysis and were confirmed for selective media (SD-TL, SD-TLH, and SD-TLH supplemented with 10 mM 3AT) after restreaking (Figure 6A). Sequence analysis identified twenty-two genes as candidate upstream transcription factors (UTFs) of *BnaMATE43b*, including six bHLH, four ERF, three bZIP, MYB, and other transcription factors (Supplementary Table S6).

To determine the appropriate conditions for one-on-one verification, control experiments were performed. The positive control (pHIS2-p53 + pGAD53m) grew normally on SD-TL, SD-TLH, and SD-TLH + 30 mM 3AT plates. In contrast, the blank control (pHIS2-BnaMATE43b-pro + pGADT7) grew on SD-TL and SD-TLH plates but failed to grow on SD-TLH plates supplemented with 30 mM 3AT. Subsequent one-on-one Y1H assays revealed that the BnaMATE43b promoter (in pHIS2) interacted with pGADT7-BnaA02g28220D, pGADT7-BnaA05g23130D, pGADT7-BnaA06g07840D, and pGADT7-BnaA08g24520D. pHIS2-BnaMATE43b-pro does not interact with pGADT7-BnaA08g16990D and pGADT7-BnaC03g65930D (Figure 6B). These results suggest that the *BnaMATE43b* promoter may interact with *BnaA02g28220D*, *BnaA05g23130D*, *BnaA06g07840D*, and *BnaA08g24520D* but not with *BnaA08g16990D* and *BnaC03g65930D*.

To further identify the UTFs of *BnaMATE43b*, four candidate factors were verified to interact with *BnaMATE43b* through luciferase assays. The Luc/Ren of D220, D130, D840, and D520 experimental groups ranged from 0.056 to 0.082, and the Luc/Ren of D520 was the highest, while that of the positive control group was 0.066. Both the experimental group and the positive control group were significantly higher than the blank control group in DCK1- (0.029) and the blank control group in DCK2- (0.014) (Figure 6C). In summary, the *BnaMATE43b* promoter could interact with *BnaA02g28220D*, *BnaA05g23130D*, *BnaA06g07840D*, and *BnaA08g24520D* with Y1H and luciferase assays.

## 3. Discussion

In plants, MATE transporters play diverse physiological roles, including the transport of secondary metabolites and xenobiotics, maintenance of iron homeostasis, Al-induced citrate efflux, and modulation of disease resistance pathways [29,30,31,32]. The *Arabidopsis* MATE transporter DETOXIFICATION 30 (DTX30) modulates the auxin contents in the root to cause elongation of the primary root and lateral roots [14]. The Al locus encoding a MATE protein was mapped and cloned in sorghum, expressed predominantly in root tips under Al stress, and promotes citrate release to detoxify Al toxicity [33]. Numerous *MATE* genes have been identified to improve Al resistance via a similar mode of citrate exudation, such as *OsFRDL4* and *OsFRDL2* in rice [34,35], *GmMATE13* in soybean [13], *HvAACT1* in barley [36], and *VnMATE1* and *VnMATE2* in rice bean [37]. In *Brassica napus*, 124 *MATE* genes were identified, with many showing differential expression between plants under heavy metal or hormone treatments when compared with control plants [28]. These results indicated that Group 2 and 3 *MATE* genes may play important roles in stress tolerance and hormone induction [28]. In our previous research, three differentially expressed *MATE* genes were detected with the integration of GWAS and RNA-seq, including *BnaMATE43b* (belonging to Group 3 *MATE* genes) and *BnaA03g30330D* adjacent to SNP *Bn-A03-p14798182* and *BnaA09g14730D* adjacent to SNP *Bn-A09-p8460525* [27]. Interestingly, only *BnaMATE43b* was differentially expressed in ASL and ATL under both 6 h vs. 0 h and 24 h vs. 0 h, and it was more downregulated in ASL than in ATL (Figure 1A) [27]. In this study, *BnaMATE43b* was cloned from ATL and overexpressed in Westar. Compared with WT, the RET and RTRL in the OE lines were increased by an average of 13.41% and 10.1% under Al toxicity stress; these results showed that the overexpression of *BnaMATE43b* could improve the Al tolerance of the OE lines (Figure 2D,E).

Numerous studies have shown that an increase in the MATE gene family increases citric acid efflux and reduces aluminum content in the root [9,10,11,12,13,32]. And, this research focuses on the antioxidant system, such as reactive oxygen species (ROS), MDA, root activity, and the relative electrical conductivity. Under abiotic stress, the levels of ROS were induced and accumulated in plants. Accumulation of excessive ROS could damage the membrane system, among others, ultimately affecting the growth and development of plants [38,39]. During stress, the content of ROS in plants could be reduced by enhancing the activity of enzymes related to the antioxidant enzyme system, which plays an important role in alleviating damage, such as SOD and POD [19,39,40]. Under Al toxicity stress, the overexpression of *GmSTOP1-3* enhanced the activities of SOD and POD, thereby improving the Al tolerance in soybean [19]. In maize, the overexpression of *ZmAT6* resulted in lower contents of ROS in the roots compared with WT, but the activity of several enzymes of the antioxidant system (SOD and POD) was increased in transgenic plants [40]. In this study, the activities of SOD and POD enzymes and root activity in the OE lines were significantly increased under Al treatment, and the content of MDA and the relative electrical conductivity decreased (Figure 3). These results suggest that the overexpression of *BnaMATE43b* could improve the Al tolerance of the OE lines by enhancing the activity of antioxidant enzyme systems and the root activity, thereby reducing the harm caused by Al toxicity.

To clarify *BnaMATE43b*-mediating Al tolerance in rapeseed, RNA-seq was conducted and found DEGs between WT and OE-3 under Al toxicity stress. KEGG enrichment analysis showed that 86 DEGs were enriched in numerous pathways, such as zeatin biosynthesis (map00908), glucosinolate biosynthesis (map00966), plant hormone signal transduction (map04075), phenylpropanoid biosynthesis (map00940), and ascorbate and aldarate metabolism (map00053) (Figure 4D, Supplementary Table S5). Based on the integration analysis of transcriptome and metabolome in soybean under Al toxicity stress, the DEGs and differentially expressed metabolites were enriched in zeatin biosynthesis, phenylpropanoid biosynthesis, and ascorbate and aldarate metabolism [8]. In addition, the DEGs related to phenylpropanoid biosynthesis in rice were highly enriched after Al stress [41], and the DEGs in *Vitis quinquangularis* were enriched in the phenylalanine metabolic pathway and the hormone signaling pathway [42]. These results suggest that *BnaMATE43b*, regulating the tolerance of rapeseed to Al toxicity, may be influenced by the expression of genes in these pathways (map00908, map00966, map04075, map00940, and map00053). Preliminary analysis of the mechanism by which BnaMATE43b regulates Al toxicity tolerance through these KEGG pathways was combined with the metabolomehese pathways [8,42]. Further determination of related important metabolites, such as phenylalanine, flavonoids, and organic acids, is carried out for validation and systematic analysis [43].

Transcription factors play a crucial role in regulating plant responses to abiotic stress [16,17,19,20]. Under Al stress, an NAC transcription factor, *VnNAR1*, regulated the metabolism of pectin in the cell wall by increasing the expression level of cell wall-associated receptor kinase 1 (WAK1), thereby affecting the Al tolerance of plants [44]. Loss of function of *ANAC017* could improve Al tolerance and reduce the root and root cell wall Al content compared with WT under Al stress, and *ANAC017* regulated the low expression of *XTH31* for decreasing the content of hemicellulose and xyloglucan to enhance the Al tolerance in mutants [45]. In addition, overexpression of *GsMYB10* and *GmSTOP1-3* could enhance the Al resistance of soybean [19,46]. In this study, to screen the upstream transcription factors of *BnaMATE43b*, the yeast cDNA library of rapeseed was constructed. Based on the library, 22 genes were obtained as the candidate UTFs of *BnaMATE43b* using Y1H, including bHLH, ERF, bZIP, and MYB transcription factors (Figure 6A, Supplementary Table S6). Six genes were randomly selected from the twenty-two candidate UTFs to one-on-one interaction validation with MATE, revealing that four of them (*BnaA02g28220D*, *BnaA05g23130D*, *BnaA06g07840D*, and *BnaA08g24520D*) could interact with the MATE promoter (Figure 6B). The results of luciferase assays were consistent with the one-on-one verification (Figure 6C). These four candidate UTFs belong to the bHLH DNA-binding protein superfamily (*BnaA02g28220D*, *BnaA06g07840D*, and *BnaA08g24520D*) and the ethylene-responsive element-binding protein family (*BnaA05g23130D*). In both *Arabidopsis* and *Stylosanthes* hairy roots, overexpression of *SgPG1* could enhance the Al resistance by changing the cell wall pectin epitopes, and the UTF *SgbHLH19* of *SgPG1* was detected using the Y1H assay, the electrophoretic mobility shift assay (EMSA), and luciferase complementation assays [47]. Due to the overexpression of *GmABR1*, when encoding an ERF transcription factor, the Al tolerance of mutants was enhanced, and the Al stress-responsive *AtMATE* genes were upregulated in mutants [21]. These results showed that three bHLH factors and one ERF factor may regulate the Al tolerance of rapeseed by mediating the expression of *BnaMATE43b*, and the function of the four UTFs under Al toxicity stress needs to be further verified.

## 4. Materials and Methods

### 4.1. Validation Expression Pattern of BnaMATE43b Based on qRT-PCR

The Al-tolerant inbred line R178 (ATL) and the Al-sensitive inbred line S169 (ASL), referred to in our previous studies, were used as materials in this study [20]. In our previous study, *BnaMATE43b* was screened by integrating GWAS and RNA-seq, and the expression patterns of *BnaMATE43b* were significantly differentially expressed between 6 h vs. 0 h and 24 h vs. 0 h in ATL and ASL, respectively. The expression patterns were then verified with quantitative real-time PCR (qRT-PCR). The primer sequences of *BnaMATE43b* and the reference *ACT7* genes [48] are shown in Supplementary Table S7 and were synthesized at Tsingke Biotech (Beijing, China). The 2^−ΔΔCt^ method was used to analyze the expression pattern. Three technical replicates were performed.

### 4.2. Generation of Transgenic Westar Plants

The full-length CDS of *BnaMATE43b* was cloned with PCR, and the cDNA template was reversed in a previous study [48]. The primer sequences of the *BnaMATE43b* CDS are shown in Supplementary Table S7. Based on pCAMBIA1301 as the basic vector, the overexpression vector of *BnaMATE43b* was constructed. Then, the overexpression vector was transformed into wild-type rape Westar by Wuhan Biorun Biosciences Co., Ltd. (Wuhan, China). PCR tests were conducted on the plants returned by the company using positive detection primers (Supplementary Table S7). The positive test plants of PCR produced 683 bp. After harvesting positive OEs of the T0 generation, the positive plants of the T1 generation and T2 generation were obtained through continuous self-fertilization. Finally, the T3 seeds were used for further experiments, and their expression patterns were detected using qRT-PCR verification.

### 4.3. Morphological and Physiological Parameters Under Al Treatment

Seeds of uniform size were selected from OE-3 and WT. Then, the seeds were sterilized in 1% hydrogen peroxide and washed with ultrapure water before spreading on the gauze cloth. The uniform and healthy rapeseed seedlings were sequentially transferred into 1/8 (3 d) and 1/4 (3 d) Hoagland’s nutrient solutions without Al treatment gradually [27]. OE-3 and WT were exposed in 1/4 Hoagland’s nutrient solutions with 0, 30, 60, 90, and 120 μmol·L^−1^ AlCl_3_ for 7 days. The appropriate concentration of Al treatment was determined based on the response of the plant’s root system. Then, the phenotypic identification and physiological index (including SOD activity, POD activity, proline content, MDA content, relative electrical conductivity, and root activity) were analyzed at an appropriate concentration for 7 days. These physiological indicators were carried out according to the kit instructions (Suzhou Grace Biotechnology Co., Ltd., Suzhou, China).

### 4.4. RNA-Seq Under Al Treatment

To further analyze the regulatory network response to Al stress after overexpression of *BnaMATE43b*, OE-3 and WT were treated with 60 µmol·L^−1^ AlCl_3_ for 0 h and 24 h. Then, the roots were quickly frozen in liquid nitrogen and used for RNA-seq by Wekemo Tech Group Co., Ltd. (Shenzhen, China). To determine which genes correlate with *BnaMATE43b* overexpression, the differentially expressed genes (DEGs) between OE-3 and WT for 0 h and 24 h were identified. DEGs were screened with |log2 fold change| > 1.0 and *p* value < 0.05. All DEGs were annotated using the following databases: NR (ftp://ftp.ncbi.nih.gov/blast/db/FASTA/ (accessed on 24 May 2024)), GO (http://geneontology.org/ (accessed on 24 May 2024)), and KEGG (https://www.genome.jp/kegg/ (accessed on 24 May 2024)). The raw read data reported in this study have been deposited in the Genome Sequence Archive (GSA) in the National Genomics Data Center under submission ID CRA034620 (https://ngdc.cncb.ac.cn/gsa/browse/CRA034620 (accessed on 9 December 2025)).

### 4.5. Construction of the Homogenized cDNA Library

R178 was used as the material for constructing the cDNA library. After six hours of treatment, the roots were collected for total RNA extraction. The extraction quality of total RNA was detected with agarose gel electrophoresis. The mRNA was separated and purified from high-quality total RNA. Then, cDNA was reversed using three-frame primers (P1-F/P4-R, P2-F/P4-R, P3-F/P4-R) with the SMART cDNA Library Construction Kit (TaKaRa, Beijing, China). Three double-stranded cDNAs were mixed, and the mixture of the three cDNA products was purified, homogenized, and small fragments were removed. The clone products obtained through homogenization and removal of small fragments were homologously recombined with pGADT7 AD Vector (TaKaRa, Beijing, China ).

The recombination plasmids were transformed into Escherichia coli, and 10 μL from this bacterial solution was diluted 10,000 times for library quality identification. Next, 100 μL from the diluted solution was spread on LB plates and cultured to calculate the total number of clones. Then, the titer of the library and the library capacity were obtained. Twenty-four clones were randomly picked from the plate as templates. PCR was performed on the bacterial liquid using the primers T7-F: TAATACGACTCACTATAGGGCGAGCGCCGCCATG and 3′AD: GTGAACTTGCGGGGTTTTTCAGTATCTAC. The positive rate of the clones and the fragment size of the library were detected with agarose gel electrophoresis.

### 4.6. Y1H Assay

To screen the upstream transcription factors (UTFs) of *BnaMATE43b*, the promoter (chrA03: 14709137~14712252) was synthesized and inserted into the pHIS2 vector as ‘bait’ for Y1H. The pHIS2-BnaMATE43b-pro construct was transferred into yeast strain Y187, and competent cells were prepared. Subsequently, the root cDNA library plasmid pGADT7-cDNA was transferred to pHIS2-BnaMATE43b-pro competent cells and cultured on SD-Leu-Trp-His solid agar plates for 3 days at 30 °C. Repeat validation of yeast-positive clones and detection to verify the reliability of positive clones. All yeast-positive clones were sequenced via high-throughput sequencing, and the sequences of clones were blasted for function annotation.

Before the point-to-point interaction verification assay, the UTFs of *BnaMATE43b*, detected by positive monoclonal sequencing or scraper sequencing, were selected for transformation verification. Three groups, namely, the experimental groups Y220, Y130, Y840, Y990, Y520, Y930, the negative control group YCK−, and the positive control group YCK+, were co-transformed according to Table 1. They were incubated at a constant temperature of 30 °C for 3 days to observe and determine whether the promoter and transcription factor have a point-to-point interaction.

### 4.7. Dual Luciferase Reporter Assay

The upstream sequences of 1582 bp (chrA03: 14709137-14712252) for the *BnaMATE43b* were synthesized and ligated into the pGreenII 0800-LUC vector. The CDSs of the candidate transcription factors *BnaA02g28220D*, *BnaA05g23130D*, *BnaA06g07840D*, and *BnaA08g24520D* were ligated into the pGreenII-62sk vector. The empty pGreenII-62sk vector was used as the negative control group, and the pGreenII-62SK-AtMYB75 was used as the positive control group. The vector plasmids were transferred to agrobacterium GV3101 (Zhuangmeng Biology, Beijing, China) using the combination (Table 2) electroconversion method, which was carried out in accordance with its operation manual.

Tobacco was cultivated for one month, until later use. Plasmid vector-transformed agrobacterium GV3101 was cultured for 2 days and then inoculated in 10 mL of YEB liquid medium (containing the corresponding resistance element) for 1 h, following which the suspended bacteria were collected. The bacteria were resuspended in a suspension of 10 mmol·L^−1^ MgCl_2_ (containing 120 μmol·L^−1^ AS); the OD600 was adjusted to about 1.0, and it was left to stand for culture. Tobacco plants in a good growth condition were selected. The bacterial solution was mixed proportionally and then injected into the lower epidermis of the tobacco leaves with a syringe without a needle and marked accordingly. The tobacco plants that had been injected were cultivated under weak light for 2 days before detection. A Dual Luciferase Reporter Assay Kit (VAzyme, Nanjin, China) was utilized in accordance with the manufacturer’s instructions, mainly including the steps of cell lysis, firefly luciferase reaction detection, and Renilla luciferase reaction detection, with three replicates for each detection. Taking Renilla luciferase (Ren) as the internal reference, the value obtained with the determination of firefly luciferase (Luc) was divided by the value of Ren, and the experimental result was Luc/Ren.

## Figures and Tables

**Table 1:** The reaction plasmid compatibility of point-to-point interaction verification.

Reaction	Transcription Factor	pHIS2 Plasmid	AD Plasmid
Y220	*BnaA02g28220D*	pHIS2-BnaMATE43b-pro	pGADT7-BnaA02g28220D
Y130	*BnaA05g23130D*	pHIS2-BnaMATE43b-pro	pGADT7-BnaA05g23130D
Y840	*BnaA06g07840D*	pHIS2-BnaMATE43b-pro	pGADT7-BnaA06g07840D
Y990	*BnaA08g16990D*	pHIS2-BnaMATE43b-pro	pGADT7-BnaA08g16990D
Y520	*BnaA08g24520D*	pHIS2-BnaMATE43b-pro	pGADT7-BnaA08g24520D
Y930	*BnaC03g65930D*	pHIS2-BnaMATE43b-pro	pGADT7-BnaC03g65930D
YCK-	—	pHIS2-BnaMATE43b-pro	pGADT7
YCK+	—	pHIS2-p53	pGAD53m

**Table 2:** Reaction plasmid compatibility of the dual luciferase reporter experiment.

Reaction	Transcription Factor	pGreenII0800-LUC Plasmid	pGreenII-62SK Plasmid
D220	BnaA02g28220D	pGreenII0800-LUC-pro	pGreenII-62SK-220D
D130	BnaA05g23130D	pGreenII0800-LUC-pro	pGreenII-62SK-130D
D840	BnaA06g07840D	pGreenII0800-LUC-pro	pGreenII-62SK-840D
D520	BnaA08g24520D	pGreenII0800-LUC-pro	pGreenII-62SK-520D
DCK1-	—	pGreenII0800-LUC-pro	pGreenII-62SK
DCK+	—	pGreenII0800-LUC-AtDFRpro	pGreenII-62SK-AtMYB75
DCK2-	—	pGreenII0800-LUC-AtDFRpro	pGreenII-62SK

## Data Availability

The data presented in this study are available in this article and the Supplementary Materials, and the original RNA-seq data presented in the study are openly available in the Genome Sequence Archive (GSA) at https://ngdc.cncb.ac.cn/gsa/browse/CRA034620 (accessed on 9 December 2025).

## Supplementary Materials

The following supporting information can be downloaded at https://www.mdpi.com/article/10.3390/plants15020338/s1, Supplementary Table S1. The data statistics of RNA-seq samples output. Supplementary Table S2. The DEGs of 0E-3 0 h vs. WT 0 h under Al toxicity treatment. Supplementary Table S3. The DEGs of 0E-3 24 h vs. WT 24 h under Al toxicity treatment. Supplementary Table S4. The DEGs enriched in GO. Supplementary Table S5. The DEGs enriched in the KEGG. Supplementary Table S6. The screening of transcription factors with the yeast one-hybrid assay. Supplementary Table S7. The primer sequences.
